# Burnout among French healthcare workers caring for individuals with profound intellectual and multiple disabilities/polyhandicap: a complementary cross-sectional and longitudinal study

**DOI:** 10.3389/fpubh.2026.1906622

**Published:** 2026-09-28

**Authors:** Houria El Ouazzani, Marie-Christine Rousseau, Any Beltran-Anzola, Sibylle Del Duca, Ilyes Hamouda, Thierry Billette de Villemeur, Pascal Auquier, Karine Baumstarck

**Affiliations:** 1 Epidemiology and Health Economy Department, Marseille Public University Hospital System, Marseille, France; 2 UR 3279, CEReSS - Research Centre on Health Services and Quality of Life, Aix Marseille University, Marseille, France; 3 Fédération des Hôpitaux de Polyhandicap et Multihandicap, San Salvadour Hospital, University Hospital of Paris, Hyères, France; 4 Clinical Research Group GRC ConCer-LD, Sorbonne University & Pierre-et-Marie-Curie University, Paris, France; 5 Department of Neuropediatrics, Armand-Trousseau Hospital, Paris, France

**Keywords:** burnout, health care workers, polyhandicap, PIMD, cohort

## Abstract

**Purpose:**

Polyhandicap, known as Profound Intellectual Multiple Disabilities, is characterized by profound intellectual impairment and severe motor deficits, leading to a high level of dependence. Caring for these individuals has specific stressors, including lack of patient feedback or recognition, frequent unfavorable clinical trajectories, and complex relationships with families. This study aimed to assess burnout among health care workers caring for persons with PIMD/polyhandicap, to identify associated factors, and to examine the stability of burnout over time within the French EVAL-PLH cohort.

**Materials and method:**

This study used complementary cross-sectional (2020) and longitudinal (2015–2020) designs. Burnout was assessed using the Maslach Burnout Inventory among health care workers caring for persons with PIMD/polyhandicap.

**Results:**

In 2020, 208 health care workers were included; 79 (38%) presented severe burnout according to the French global MBI scoring. Higher emotional exhaustion was associated with self-reported mood disorders, use of mood disorder medication, employment in residential facilities, and more frequent avoidance coping. Higher depersonalization was associated with younger age and more frequent avoidance coping. Lower personal accomplishment was associated with single status and less frequent positive-thinking coping. In the longitudinal subsample (*n* = 58), 21 HCWs (37%) experienced severe burnout in 2015, of whom 17 (81%) remained severely burned out in 2020; Cohen’s kappa indicated moderate agreement between T1 and T2 burnout classifications (κ = 0.539, *p* < 0.001), while McNemar’s test showed no significant change in burnout status over time (*p* = 0.267).

**Conclusion:**

Burnout is highly prevalent among health care workers caring for persons with PIMD/polyhandicap and shows partial temporal stability. Addressing modifiable associated factors, such as coping strategies and mental health support, should be a key focus of intervention programs to sustain the workforce and ensure continuity and quality of care for this highly dependent population.

**Trial registration:**

Identifier, NCT02400528.

## Introduction

Burnout is a form of work-related stress that can be defined as a state of mental, emotional, and physical exhaustion resulting from chronic, unmanaged work-related stress (1). Although burnout affects the general working population, studies suggest a higher prevalence among healthcare workers (HCWs) (2, 3), with more than one third of health professionals worldwide suffering from burnout across various professions (nurses, midwives, and physicians) (4–7). Multiple factors potentially associated with burnout among HCWs, including high workload and time pressure, lack of positive feedback, difficulty managing stressful situations, and exposure to hierarchical pressure and contradictory instructions (7). The consequences are considerable: HCWs’ health and quality of life deteriorate, sometimes with risks of suicide and addiction; quality of care may decline, with increased malpractice and strained relationships with patients and families; and healthcare organizations face higher absenteeism, turnover, and weakened teams (4, 7, 8).

More recently, a systematic review of burnout among staff working in services for adults with intellectual and developmental disabilities (IDD) examined organizational and individual factors of burnout (9). Overall, staff supporting adults with IDD showed lower emotional exhaustion, depersonalization, and personal accomplishment than normative samples from other human services. While this review focuses on staff supporting adults with IDD in general, much less is known about burnout among healthcare professionals caring for persons with Profound Intellectual and Multiple Disabilities (PIMD)/polyhandicap. PIMD is defined as a severe and complex chronic condition resulting from early brain damage and characterized by both profound intellectual impairment and severe motor deficits. This clinical presentation leads to extremely restricted autonomy and communication and, consequently, a high level of dependency (10, 11). When the disorder affects an immature brain, the term of polyhandicap is used; polyhandicap, as a subgroup of PIMD, includes the most severe cases due to the early onset of the brain damage. Recently, the Ithaca European Reference Network for congenital malformations and rare intellectual disabilities has adopted the combined term PIMD/polyhandicap (11).

Caring for these individuals has several specific features: healthcare workers receive little or no feedback, complaint, or recognition from the patient (12, 13); the clinical picture is often unfavorable, with numerous comorbidities and no expectation of improvement (14, 15); and families may be absent, distant, or distrustful, complicating relationships with HCWs (16). Supporting these patients can be compared to a form of long-term palliative care involving multiple professionals, with the aim of treating and preventing comorbidities while stimulating communication and cognitive abilities to improve quality of life. In France, these HCWs mainly work in specialized rehabilitation centers or residential facilities, which represent the two main care modalities for persons with PIMD/polyhandicap.

Assessing burnout and understanding the specific factors associated with it among these HCWs will enable the development of effective interventions to improve their well-being and work experience. Our study is grounded in Maslach’s three-component model of burnout, conceptualized as emotional exhaustion, depersonalization, and reduced personal accomplishment, and operationalized by the Maslach Burnout Inventory (MBI) (9). This model is consistent with broader frameworks of occupational stress, such as the Job Demands–Resources model and transactional stress and coping theories, which consider how job demands, resources, and coping strategies shape the three dimensions of burnout in this specific care context.

We hypothesized that burnout in HCWs caring for persons with PIMD/polyhandicap would be associated with specific sociodemographic, professional, health-related, and psycho-behavioral characteristics. More specifically, we expected burnout to vary according to coping strategies, mood symptoms, and selected demographic characteristics. Building on this theoretical background, the present study is hypothesis-driven rather than purely exploratory; we therefore used the MBI not only as a descriptive measure of burnout, but also as an operationalization of Maslach’s model to examine these associations. The aims of this study were to assess burnout among these healthcare workers, to identify associated factors of burnout, and to analyze change over time in burnout.

## Materials and methods

### Design of the study

The study is included in the French EVALuation PoLyHandicap (EVAL-PLH) cohort. At present, three evaluation time points are planned: 2015, 2020, and 2025 (ongoing). In this paper, cross-sectional and longitudinal designs were combined. The cross-sectional analysis used data from the second evaluation time point (2020), and the longitudinal analysis used the subsample of participants assessed at both time points (2015 and 2020). At the time of analysis, the 2025 data were not available. Because of the limited size of this longitudinal subsample, the longitudinal analyses were considered exploratory and hypothesis-generating. The cohort was designed to assess the health status of people with PIMD/polyhandicap, as well as the life experiences of their informal and institutional caregivers (healthcare workers) (17). The persons with polyhandicap were defined by the combination of a severe motor deficit (tetraparesis, hemiparesis, paraparesis, extra pyramidal syndrome, cerebellar syndrome, and neuromuscular disorders), profound intellectual disability (non-evaluable intellectual quotient or IQ < 40), extreme dependence in daily life (Functional Independence Measure score < 55), and reduced mobility (Gross Motor Function Classification Scale level > III).

### Participants

The selection criteria of the HCWs were being at least 18 years old, caring for patients with PIMD/polyhandicap, and agreeing to participate. The healthcare workers were recruited from four specialized rehabilitation centers (Hendaye Hospital, Armand Trousseau, La Roche Guyon Hospital, and San Salvadour Hospital; Assistance Publique des Hôpitaux de Paris) and eight residential facilities located in two French regions (CESAP—Comités d’Études, d’Education et de Soins Auprès des Personnes Polyhandicapées, in Ile-de-France and Hauts-de France). The following professional categories were included: nurses, physicians, psychometricians, occupational therapists, nursing assistants, and educators. No formal sample size calculation was performed, as this is a descriptive study primarily aimed at characterizing the population and assessing the prevalence of severe burnout. However, assuming an expected prevalence of severe burnout greater than 30% with a confidence interval of up to 15% (± 7.5%), and a bilateral α risk of 5%, the inclusion of at least 155 HCWs would be sufficient (PASS 2024, version 24.0.2).

### Study procedures and data collection

A specific booklet was distributed to each eligible healthcare worker. HCWs who agreed to participate were asked to return the anonymized booklet in a prepaid envelope. At T1, the questionnaire was distributed on site by a dedicated research assistant, together with a prepaid envelope allowing immediate free postal return. Each booklet contained only a coded identifier, while the name–identifier correspondence was retained locally by the research assistant and was not accessible to the coordinating center. A standardized, non-individualized reminder procedure was implemented 2 weeks later. Returned questionnaires were received by the coordinating center, which handled only coded data. At T2, the local research assistant updated the correspondence list and reassigned the same identifier to HCWs who had already participated at T1; the same procedure was then followed.

The data included:

- Sociodemographic characteristics: age, gender, marital status (single vs. not single), having children (yes vs. no), level of education (< 12 vs. ≥12 years), and self-perceived financial situation (comfortable vs. financial difficulties).
- Health data: presence of chronic diseases (yes vs. no) and hospitalization episode in the past 12 months (yes vs. no).
- Professional data: care structure (specialized rehabilitation centers vs. residential facilities), professional category (technical—nurses, physicians, psychometricians, and occupational therapists—vs. non-technical care—nursing assistants and educators), work schedule (full-time vs. part-time), seniority in caring people with polyhandicap (years), and seniority in the current center (years).
- Psycho-behavioral data including: (i) Self-reported mood disorders, assessed using an *ad hoc* Likert-type scale ranging from 1 (absence) to 10 (maximal level of anxiety/depression), which has shown good correlation with validated questionnaires such as the Hospital Anxiety and Depression Scale (18). This brief measure was retained to limit respondent burden; (ii) Coping, assessed using the French version (19) of the Brief COPE (Coping Orientation to Problems Experienced) inventory, a 28-item scale (20) assessing the use of four coping strategies (21): social support, problem solving, avoidance, and positive thinking. The higher the score (ranging from 0 to 100), the more likely the strategy is to be used.
- Burnout: the burnout was assessed using the Maslach Burnout Inventory (MBI) (22–25). This 22-item tool characterizes burnout by three dimensions: (i) increased emotional exhaustion, (ii) increased depersonalization, and (iii) reduced personal accomplishment. A validated French version of the MBI, widely used in clinical work on burnout among healthcare professionals, was employed in this study (26). The inventory yields scores for 3 dimensions (emotional exhaustion ‘ee’, depersonalization ‘dep’, and personal accomplishment ‘pa’) and a global burnout score (‘ee’ + ‘dep’ – ‘pa’) ranging from −48 (low) to 90 (high level of burnout). The French version of the tool has been used widely in French clinical and occupational health research and has shown acceptable psychometric properties in French healthcare samples (27), while the international literature continues to debate whether a global score should be used alongside or instead of the three dimensions (28). Internal consistency in the present sample was good for emotional exhaustion and reduced personal accomplishment, and lower for depersonalization, with Cronbach’s alpha values of 0.89, 0.50, and 0.78 at T1, and 0.86, 0.38, and 0.65 at T2, respectively. In line with French proposals for summarizing the MBI into a single score, three categories were defined (26): severe, moderate, and low. This categorization is consistent with French clinical work on burnout among healthcare professionals. In this study, dimension-specific analyses were conducted as the primary analyses. Complementary (sensitivity) analyses compared participants classified as having severe burnout with those not classified as having severe burnout (i.e., those with moderate or low burnout levels).

### Outcomes

- Prevalence of severe burnout among institutional HCWs caring for people with PIMD/polyhandicap.
- Potential factors associated with burnout: we examined sociodemographic variables, variables related to health status, variables related to professional life, and psycho-behavioral variables. These variables are detailed in the previous section.
- Analysis of burnout changes over time: the study included an analysis of burnout stability over time from 2015 to 2020. This approach allows for the assessment of the temporal stability of burnout among participants.

### Biases

In this multicenter study using an anonymous booklet with a prepaid return envelope and reminders, several biases were controlled: non-response and voluntary response bias were mitigated through reminders, thereby promoting more representative participation by healthcare professionals; social desirability bias was reduced by ensuring anonymity (no interviewer, return by mail), encouraging more honest responses about burnout; and potential convenience sampling bias was minimized through multicenter recruitment, improving generalizability. These measures strengthen both the internal and external validity of the prevalence estimate, although they do not control for all biases, such as issues related to temporality or residual confounding factors.

### Statistical analysis

Statistical significance was defined as *p* < 0.05. Data were analyzed using SPSS software, version 20.0. No data imputation was performed. The normality of the distributions was systematically tested using the Shapiro–Wilk (SW) test, and results were presented accordingly, as mean and standard deviation or median and interquartile range. The final analytical sample was defined as participants who returned the questionnaire and completed the MBI, as the main analyses relied on burnout classification derived from this instrument.

From the cross-sectional design, descriptive analysis was performed on the characteristics of the sample: quantitative variables were described by mean and standard deviation or median and interquartile range according to the SW test, and qualitative variables were described by numbers and percentages. Proportions of burnout levels were provided with the 95% confidence intervals (CI). Global and dimension scores of the MBI were described. To determine factors associated with the 4 MBI scores (emotional exhaustion, depersonalization, personal accomplishment scores, and global score), multivariate models (linear regression) were performed (variable selection based on univariate analysis, *p* < 0.05). Results were provided as standardized betas (β). Dimension-specific analyses were considered the primary analyses. The three dimension scores reflect specific components of burnout, whereas the global score captures overall burnout severity. Predefined sensitivity analyses were performed to determine factors associated with severe burnout. Bivariate analyses were performed using chi2 or Fisher’s exact tests for qualitative variables and Student t test for quantitative variables. Multivariate models (logistic regressions) were performed. Multiple model-building strategies for variable selection were based on: (i) Model 1, using a selection of variables based on univariate analyses with *p* < 0.05; (ii) Model 2, using a selection of variables based on univariate analyses with *p* < 0.25; (iii) Model 3, using the same selection as Model 1 but additionally including age and sex (two factors associated with burnout); and (iv) Model 4, using the same selection as Model 3 but additionally including marital status and having children (factors associated with burnout). Models 1–4 are reported as predefined sensitivity analyses. These sensitivity analyses were designed to assess the robustness of the findings under alternative variable selection and adjustment strategies. Results were provided as odds ratio (OR) and the 95% CI. Model fit was assessed using the Hosmer–Lemeshow goodness-of-fit test, with a *p*-value greater than 0.05 indicating an adequate fit between observed and predicted values.

From the longitudinal design, temporal changes in binary burnout status were explored using McNemar’s test, while Cohen’s kappa was used to assess the concordance of categorical burnout classification between T1 and T2. Sankey diagrams were additionally used to visualize individual transitions over time. To identify the factors collected in 2015 associated with severe burnout in 2020, only univariate analysis were performed. Due to the small sample size, no multivariate adjustment was carried out.

## Results

### Cross sectional design

From March 2020 to December 2021, 233 HCWs returned their booklets out of 363 eligible persons (response rate of 57%). Of these, 208 HCWs completed the MBI questionnaire and were included in this analysis. The flow chart is presented in Figure 1. Comparison of eligible professionals by age and gender showed no significant differences. Participants were mainly women (82%), with a mean (± standard deviation SD) age was 41.8 ± 11.0 years. More than 60% worked in specialized rehabilitation centers. The mean (± SD) duration of experience in the care of individuals with polyhandicap was 11.7 ± 9.4 years. All descriptive data are summarized in Table 1. The mean (SD) global MBI score was −11.0 (14.3) and the median (IQR) global MBI score was −12 (−22;-3). According to the French global MBI scoring (categorical severe/moderate/low burnout classification), severe burnout was present in 79 HCWs (38%), while 129 HCWs (62%) presented with non-severe burnout, including 36.5% with moderate and 25.5% with low levels of burnout.

**Figure 1:**
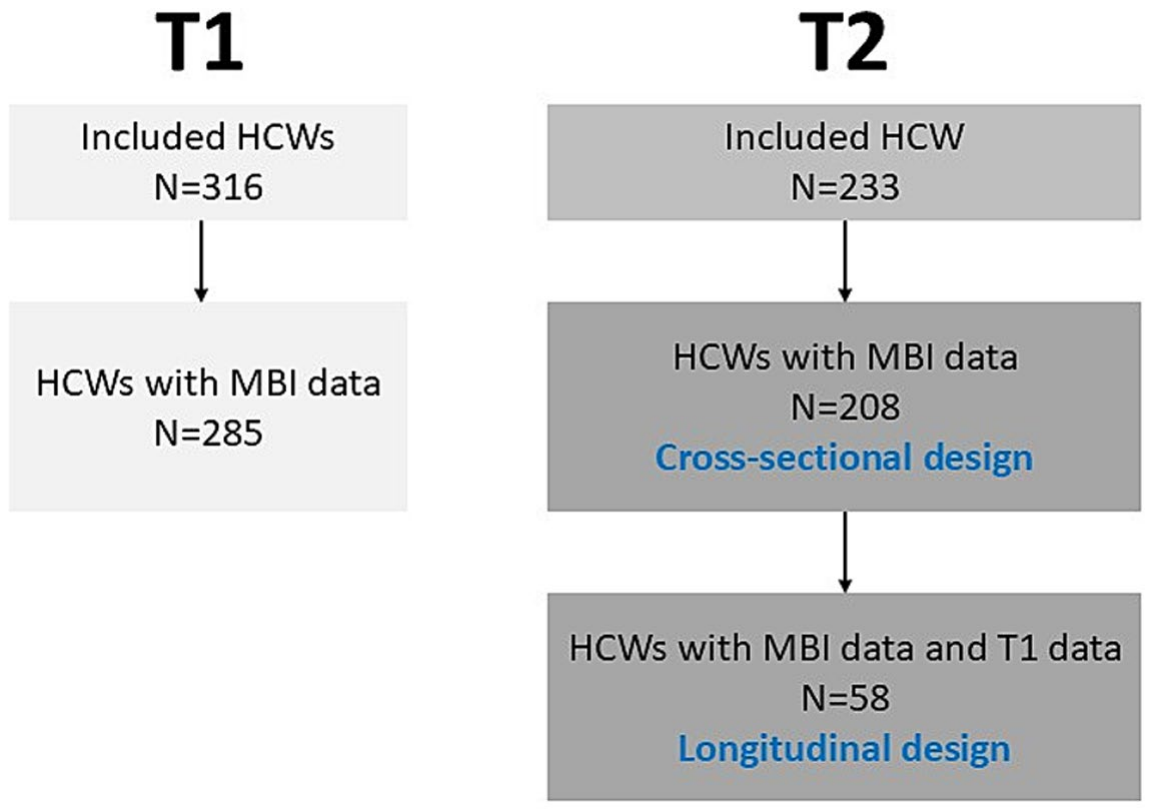
Flow chart.

**Table 1:** Characteristics of the 208 healthcare workers included in the cross-sectional study.

Variables	Modalities	N (%) M ± SD	MD N
Sociodemographics			
Age (years)		41.8 ± 11.0	0
Gender	Male	37 (17.8)	0
	Female	171 (82.2)	
Marital status	Single	65 (31.7)	3
	No single	140 (68.3)	
Having children	No	56 (26.9)	0
	Yes	152 (73.1)	
Educational level	< 12 years	69 (33.5)	2
	≥12 years	137 (66.5)	
Self-perceived financial situation	Comfortable	130 (63.7)	4
	Difficult	74 (36.3)	
Personal health			
Chronic diseases	No	127 (61.4)	1
	Yes	80 (38.6)	
Hospitalization episode	No	185 (89.4)	1
	Yes	22 (10.6)	
Professional characteristics			
Care structure (*N* = 208)	Specialized rehabilitation center	139 (66.8)	0
	Residential facility	69 (33.2)	
Professional category*	Technical Care	36 (17.5)	2
	Non-technical Care	170 (82.5)	
Work schedule	Full-time	172 (82.7)	0
	Part-time	36 (17.3)	
Seniority in polyhandicap care (years)		11.7 ± 10.0	3
Seniority in current center (years)		10.0 ± 8.8	
Psychobehavorial data			
Mood disorders (score)**		5.2 ± 2.6	1
Coping (Briefcope)***	Social support	42.4 ± 21.1	4
	Problem solving	57.7 ± 22.6	5
	Avoidance	26.1 ± 11.7	6
	Positive thinking	52.9 ± 18.6	6
Burnout (MBI)	Severe Burnout	79 (38)	0
	Global score+	−11.0 ± 14.3	0
	Emotional exhaustion+	24.7 ± 10.1	0
	Depersonalization+	9.2 ± 4.1	0
	Personal accomplishment++	44.9 ± 6.6	0

N (%) number (percent); M ± SD, mean ± standard deviation; MD, missing data. MBI, Maslach Burnout Inventory. *Professional category: Technical Care (nurses, physicians, psychometricians, and occupational therapists) and Non-technical Care (nursing assistants and educators). **Self-reported mood disorders: Scores ranging 0 to 10, the higher the score the higher anxiety-depression disorder. ***Coping strategies (Brief COPE): Scores ranging 0 to 100, the higher the score the higher coping strategy is used. ^+^The higher the score the higher burnout level, ^++^ the lower the score the higher burnout level.

We studied factors associated with MBI dimension scores. A multivariate analysis, including variables found to be significant in the univariate analysis, identified the following associations: 1. Higher emotional exhaustion score (greater burnout): higher levels of self-reported mood disorders, use of mood disorder medication, working in residential facilities, and more frequent use of avoidance coping strategies; 2. Higher depersonalization score (higher burnout): younger age and more frequent use of avoidance coping strategies; 3. Lower personal accomplishment (higher burnout): being single and less frequent use of positive thinking coping strategies. All details are summarized in Table 2. As sensitivity analysis, we studied factors associated with global MBI score and severe burnout (according to the French categorical severe/moderate/low burnout classification derived from the global score). Higher global MBI score (greater burnout): higher levels of self-reported mood disorders, more frequent use of avoidance coping strategies, and less frequent use of positive thinking coping strategies. Corresponding details are provided in Supplementary Table 1. After adjustment, across all four models, the independent factors associated with severe burnout (severe vs. non severe) were having been hospitalized in the past year, those reporting higher self-reported mood disorder scores, and those using avoidance coping strategies. All details are summarized in Table 3.

**Table 2:** Determinants of constitutive dimensions of burnout.

Variables	Modalities	Emotional exhaustion				Depersonalization				Personal accomplishment			
		M ± SD/R	p^+^	β	p^++^	M ± SD/R	p^+^	β	p^++^	M ± SD/R	p^+^	β	p^++^
1. Sociodemographics													
Age		0.017	0.812			−0.165	**0.017**	−0.15	**0.030**	−0.005	0.938		
Gender	Male (Ref)	23.6 ± 10.3	0.476			10.0 ± 4.6	0.185			45.1 ± 7.3	0.814		
	Female	25.0 ± 10.3				9.0 ± 4.0				44.9 ± 6.4			
Marital status	Single (Ref)	26.1 ± 9.2	0.136			8.7 ± 4.1	0.341			46.4 ± 5.9	**0.025**	−0.15	**0.024**
	No single	23.8 ± 10.2				9.3 ± 4.1				44.2 ± 6.8			
Having children	No (Ref)	27.0 ± 10.0	**0.048**	−0.10	0.064	9.7 ± 4.5	0.269			45.3 ± 5.6	0.612		
	Yes	23.9 ± 10.0				9.0 ± 4.0				44.8 ± 6.9			
Educational level	<12 years (Ref)	25.6 ± 10.9	0.431			8.8 ± 3.8	0.438			44.4 ± 7.0	0.491		
	≥12 years	24.4 ± 9.7				9.3 ± 4.3				45.1 ± 6.3			
Person with handicap at home	No (Ref)	24.5 ± 10.1	0.267			9.0 ± 4.1	0.084			44.8 ± 6.6	0.567		
	Yes	27.6 ± 10.1				11.0 ± 4.4				45.9 ± 6.1			
Self-perceived financial status	Comfortable (Ref)	23.4 ± 9.5	**0.005**	0.04	0.516	9.1 ± 4.1	0.885			45.4 ± 6.7	0.247		
	Difficult	27.5 ± 10.6				9.2 ± 4.3				44.3 ± 6.3			
2. Personal health													
Chronic diseases	No (Ref)	23.0 ± 9.3	**0.001**	0.09	0.095	9.1 ± 4.1	0.888			45.0 ± 6.4	0.661		
	Yes	27.6 ± 10.7				9.2 ± 4.1				44.6 ± 6.9			
Hospitalization episode	No (Ref)	24.7 ± 10.3	0.918			9.1 ± 4.0	0.482			45.1 ± 6.4	0.079		
	Yes	25.0 ± 8.7				9.9 ± 4.9				42.5 ± 7.4			
Mood disorders medications	No (Ref)	22.8 ± 8.7	**<0.001**	0.13	**0.034**	8.9 ± 4.0	**0.031**	0.09	0.228	45.0 ± 6.3	0.665		
	Yes	33.4 ± 11.4				10.5 ± 4.6				44.4 ± 7.6			
3. Professional characteristics													
Care structure*	SRC (Ref)	23.2 ± 9.3	**0.002**	0.15	**0.008**	9.4 ± 4.3	0.225			44.4 ± 6.6	0.090		
	RF	27.7 ± 11.0				8.7 ± 3.7				46.0 ± 6.3			
Professional category**	Technical care (Ref)	24.1 ± 9.2	0.691			9.4 ± 4.0	0.694			45.6 ± 6.5	0.474		
	Non-technical care	24.9 ± 10.3				9.1 ± 4.1				44.8 ± 6.6			
Work schedule	Full-time (Ref)	25.0 ± 10.0	0.364			9.0 ± 4.1	0.280			45.2 ± 6.4	0.159		
	Part-time	23.3 ± 10.4				9.8 ± 4.3				43.5 ± 7.4			
Seniority in polyhandicap care (years)		−0.039	0.577			−0.058	0.411			−0.042	0.545		
Seniority in current center (years)		−0.016	0.820			−0.089	0.207			−0.048	0.498		
Mood disorders (score)***		0.631	**<0.001**	0.45	**<0.001**	0.178	**0.010**	0.08	0.311	−0.030	0.670		
Coping strategies****	Social support	0.093	0.183			−0.033	0.634			0.117	0.097		
	Problem solving	−0.018	0.804			−0.002	0.980			0.263	**<0.001**	0.15	0.051
	Avoidance	0.299	**<0.001**	0.16	**0.006**	0.271	**<0.001**	0.22	**0.002**	−0.033	0.645	-	-
	Positive thinking	−0.176	**<0.012**	−0.06	0.322	0.022	0.757	-	-	0.287	**<0.001**	0.21	**0.007**

M ± SD: mean ± standard deviation. R: correlation coefficient. Bold values: *p*-values< 0.05. ^+^ *p*-value univariate analysis; ^++^ *p*-value multivariate analysis. Linear regression: β standardized beta. *Care structure: SRC, Specialized rehabilitation center; RF, Residential facility. **Professional category: Technical Care (nurses, physiatrists, psychometricians, or occupational therapists), Non-technical Care (nurse aids or educators). ***Self-reported mood disorders: Scores ranging 0 to 100, the higher the score the higher anxiety-depression disorder. ****Coping strategies (Brief COPE): Scores ranging 0 to 100, the higher the score the higher coping strategy is used.

**Table 3:** Factors associated with severe burnout.

Variables	Modalities	Severe burnout	No severe burnout									
		*N* = 79	*N* = 129		Model 1		Model 2		Model 3		Model 4	
		N (%) M ± SD	N (%) M ± SD	p^+^	OR (95%CI)	p^++^	OR (95%CI)	p^++^	OR (95%CI)	p^++^	OR (95%CI)	p^++^
Age (years)		41.6 ± 10.9	41.9 ± 11.1	0.834					1.00 (0.97–1.04)	0.750	1.01 (0.97–1.05)	0.711
Gender	Male (Ref)	15 (19.0)	22 (17.1)	0.723					0.48 (0.19–1.25)	0.134	0.41 (0.15–1.17)	0.095
	Female	64 (81.0)	107 (82.9)									
Marital status	Single (Ref)	25 (32.5)	40 (31.2)	0.856							1.41 (0.63–3.15)	0.398
	No single	52 (67.5)	88 (68.8)									
Having children	No (Ref)	24 (30.4)	32 (24.8)	0.379							0.96 (0.35–2.62)	0.931
	Yes	55 (69.6)	97 (75.2)									
Educational level (years)	<12 (Ref)	28 (35.4)	41 (32.3)	0.640								
	≥12	51 (64.6)	86 (67.7)									
Self-perceived financial situation	Comfortable (Ref)	42 (53.8)	88 (69.8)	**0.021**	1.09 (0.54–2.24)	0.802	1.08 (0.51–2.27)	0.845	1.08 (0.53–2.23)	0.826	1.09 (0.52–2.27)	0.821
	Difficult	36 (46.2)	38 (30.2)									
Chronic diseases	No (Ref)	40 (50.6)	87 (68.0)	**0.013**	1.80 (0.89–3.63)	0.100	2.17 (1.01–4.62)	0.046	1.92 (0.90–4.12)	0.093	2.29 (1.04–5.05)	**0.040**
	Yes	39 (49.4)	41 (32.0)									
Hospitalization episode	No (Ref)	66 (83.5)	119 (93.0)	**0.033**	4.29 (1.36–13.49)	**0.013**	5.88 (1.72–20.06)	**0.005**	4.21 (1.31–13.52)	**0.016**	3.60 (1.08–12.07)	**0.038**
	Yes	13 (16.5)	9 (7.0)									
Care structure*	SRC (Ref)	51 (64.6)	88 (68.2)	0.586								
	RF	28 (35.4)	41 (31.8)									
Professional category**	Technical (Ref)	13 (16.5)	23 (18.1)	0.761								
	Non-technical	66 (83.5)	104 (81.9)									
Work schedule	Full-time (Ref)	64 (81.0)	108 (83.7)	0.616								
	Part-time	15 (19.0)	21 (16.3)									
Seniority in polyhandicap care (years)		10.7 ± 8.7	12.3 ± 9.8	0.221			1.00 (0.93–1.09)	0.917				
Seniority in current center (years)		9.1 ± 7.8	10.6 ± 9.4	0.233			1.03 (0.95–1.12)	0.455				
Mood disorders (score)***		6.6 ± 2.6	4.3 ± 2.3	**<0.001**	1.41 (1.20–1.64)	**<0.001**	1.43 (1.23–1.67)	**<0.001**	1.43 (1.22–1.67)	**<0.001**	1.47 (1.25–1.72)	**<0.001**
Coping ****	Social support	42.4 ± 23.5	42.4 ± 19.5	0.982								
	Problem solving	53.3 ± 22.6	60.4 ± 22.2	**0.030**	0.99 (0.97–1.01)	0.125	0.99 (0.97–1.01)	0.318	0.99 (0.97–1.01)	0.174	0.99 (0.97–1.01)	0.294
	Avoidance	29.8 ± 14.0	23.8 ± 9.4	**<0.001**	1.03 (1.01–1.08)	**0.020**	1.04 (1.01–1.08)	**0.010**	1.03 (1.01–1.06)	**0.023**	1.03 (1.01–1.06)	**0.045**
	Positive thinking	48.4 ± 18.4	55.6 ± 18.2	**0.007**	0.99 (0.97–1.01)	0.447	0.99 (0.96–1.01)	0.346	0.99 (0.97–1.12)	0.329	0.98 (0.96–1.01)	0.207
Variable selection					Univariate *p* < 0.05		Univariate *p* < 0.25		Model 1 + age + sex		Model 3 + marital status + children	
Hosmer–Lemeshow goodness-of-fit test#					Chi2 4.8, df 8, *p* = 0.78		Chi2 3.8, df 8, *p* = 0.87		Chi2 4.3, df 8, *p* = 0.83		Chi2 6.8, df 8, *p* = 0.56	

N (%) number (percent). M ± SD, mean ± standard deviation. Bold values: *p*-values< 0.05. ^+^*p*-value univariate analysis; ^++^*p*-value multivariate analysis. Logistic regression: OR (95%CI) Odds Ratio (95% confidence interval). *Care structure: SRC, Specialized rehabilitation center; RF, Residential facility. **Professional categories: Technical care (nurses, physicians, psychometricians, and occupational therapists), Non-technical care (nursing assistants and educators). ***Self-reported mood disorders: Scores ranging 0 to 10, the higher the score the higher anxiety-depression disorder. ****Coping strategies (Brief COPE): Scores ranging 0 to 100, the higher the score the higher coping strategy is used. #Hosmer–Lemeshow goodness-of-fit test: *p*-value greater than 0.05 indicating an adequate fit between observed and predicted values.

### Longitudinal design

A total of 58 HCWs (Figure 1) were assessed at both evaluation times, T1 (2015) and T2 (2020). The characteristics of this subsample are presented in Supplementary Table 2. Three factors were found to be associated with severe burnout (according to the French global MBI scoring) at 5 years: male gender, and less frequent use of problem-solving and positive thinking coping strategies (Supplementary Table 3).

Analysis of burnout change over time in this subsample showed that 21 HCWs (37%) experienced severe burnout in 2015, of whom 17 (81%) continued to present severe burnout in 2020. Conversely, 9 HCWs (approximately 25%) who did not exhibit severe burnout in 2015 developed it by 2020. The Cohen’s kappa coefficient was 0.539 (*p* < 0.001), indicating moderate agreement between T1 and T2 burnout classifications. McNemar’s test was not significant (exact *p* = 0.267), suggesting that changes in burnout status over time were not statistically significant in the subsample. These results are illustrated in Figure 2 with a Sankey diagram (created with SankeyMATIC), providing a descriptive overview of transitions over time.

**Figure 2:**
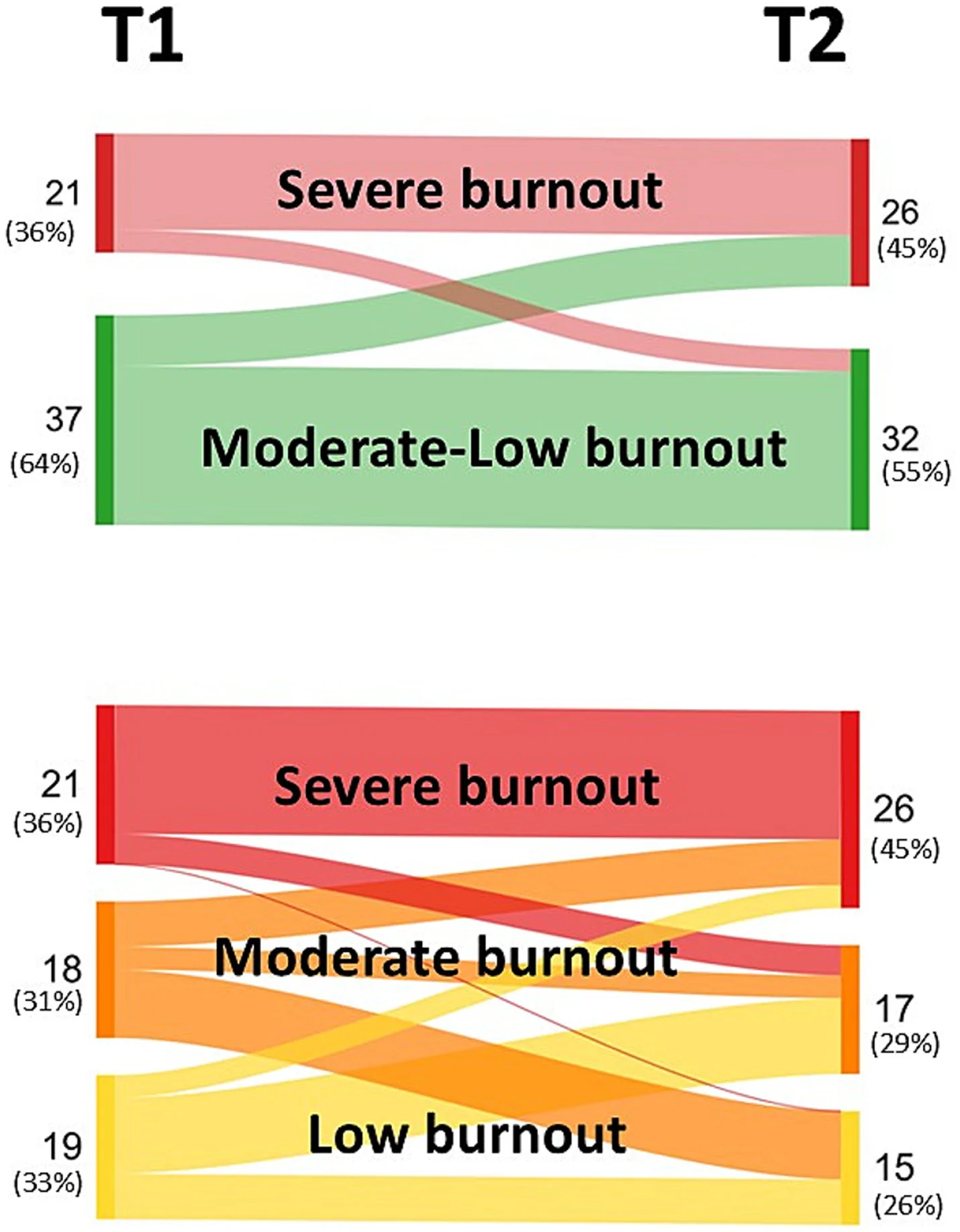
Evolution of burnout among the subsample of healthcare workers assessed in 2015 (T1) and 2020 (T2) of the EVAL-PLH study (Sankey diagram).

## Discussion

This study is the first to assess burnout among a large sample of French healthcare workers (HCWs) caring for individuals with PIMD/polyhandicap, and one particularly concerning finding is that 38% of professionals reported severe burnout according to the French global MBI scoring and classification approach. Burnout has also been reported as a substantial concern among healthcare workers in several specialties, particularly in specialties such as gynecology–obstetrics (6), oncology (29), emergency medicine (30, 31), and intensive care (32). The recent systematic review of burnout among staff working in services for adults with intellectual and developmental disabilities reported burnout scores that differed from normative human services data across the three dimensions, with lower emotional exhaustion and depersonalization and lower personal accomplishment (9). Comparisons with our findings should nevertheless be interpreted cautiously. Although our dimension-specific analyses similarly examined emotional exhaustion, depersonalization, and personal accomplishment, direct comparisons are limited by differences in study populations, settings, psychometric instruments, scoring procedures, and study designs. Moreover, our complementary analysis of severe burnout, based on a global MBI score, is not directly comparable with the dimension-specific findings reported by Baker et al. Combining the three MBI dimensions into a single score may obscure heterogeneous burnout profiles and potentially divergent associations across dimensions. These findings underscore the need for healthcare institutions and leadership teams to pay close attention to burnout among HCWs caring for persons with PIMD/polyhandicap. Addressing burnout is important, as it is associated with staff well-being and with the quality of patient care (33, 34). Previous work suggests that targeted interventions, including art therapy, cognitive-behavioral therapy, and relaxation-based techniques such as mindfulness and yoga, have been proposed to address burnout among healthcare professionals and may inform future preventive strategies in this population (35–38).

Unfortunately, our analyses did not identify a clearly distinguishable demographic or occupational profile associated with burnout. Variables such as age, gender, workplace setting, and years of experience did not consistently distinguish individuals most likely to experience burnout. This finding is broadly consistent with the systematic review by Baker et al., in which associations between burnout and individual staff characteristics were generally weak or of small magnitude. Nevertheless, the dimension-specific analyses indicated that some associations differed across the MBI dimensions, supporting the value of considering burnout as a multidimensional construct. Younger age was associated with higher depersonalization, whereas employment in residential facilities was associated with higher emotional exhaustion. These findings are broadly consistent with Baker et al., who reported that associations with staff and service characteristics could vary across MBI dimensions; however, such associations were generally weak and heterogeneous across studies (9). The presence of self-reported mood disorders—anxiety and/or depression—was associated with severe burnout in our sample, consistent with findings from previous studies (39). Baker et al. did not examine other indices of poor psychological wellbeing as potential correlates of burnout because of potential circularity and conceptual overlap between burnout and psychological distress. This overlap should therefore be considered when interpreting the association observed in our study. Another promising direction for future research would be the exploration of coping strategies, as some coping patterns may be associated with lower levels of burnout.

In this regard, several studies have demonstrated the importance of coping strategies in relation to quality of life and satisfaction indicators (40–42). Research suggests that the use of positive coping strategies, such as positive thinking and problem-solving, is more frequently associated with satisfactory experiences (43–47). Conversely, coping strategies based on avoidance or excessive reliance on social support tend to be linked with less favorable outcomes (43–45). In our dimension-specific analyses, avoidance coping was associated with higher emotional exhaustion and depersonalization, whereas less frequent positive-thinking coping was associated with lower personal accomplishment. Although Baker et al. did not synthesize these specific coping strategies, they reported weak associations between psychological acceptance or mindfulness and MBI dimensions. These findings support further investigation of coping-related resources in this population (9). Building on this line of research, targeted interventions have been developed to promote positive coping mechanisms and reduce the use of maladaptive strategies (48).

Our next finding provides a more encouraging perspective. According to the French global MBI scoring, severe burnout rates in 2020 were comparable to those observed in the 2015 sample (38.0% vs. 38.6% (17)). During the COVID-19 pandemic, healthcare teams faced numerous challenges that affected their workload and daily practice, including visitor restrictions, staffing shortages due to infections or isolation, and heightened anxiety about infection (49, 50). The present study was not designed to assess the effect of these pandemic-related circumstances on burnout. However, our results indicate that professionals who had experienced severe burnout before the pandemic largely (81%) remained classified as such during the pandemic. Because the second assessment was conducted during the COVID-19 pandemic, we cannot exclude that some participants may have experienced temporary changes in work assignment, including redeployment to COVID-related clinical activities, which may have influenced the observed results.

When analyzing the subsample assessed in both 2015 and 2020, we were unable to identify any significant associated factors with burnout. In this study, men appeared to be more prone to burnout, contrary to what has been reported in previous research (51). It is likely that studies involving larger sample sizes would provide greater statistical power to identify profiles of individuals at higher risk.

### Strengths and limitations

To our knowledge, this is the first study to investigate burnout among healthcare workers caring for individuals with PIMD/polyhandicap, using data from the French national EVAL-PLH cohort. The cross-sectional design provides an initial estimate of burnout prevalence and a preliminary overview of factors associated with burnout in this specific population of HCWs.

The sample size remained moderate, particularly for the subgroup of HCWs who participated in both data collection phases. One possible explanation is that these professionals experience high rates of absenteeism and turnover, which may have contributed to loss to follow-up. Future studies should seek to investigate the underlying reasons for these dropouts in order to confirm this hypothesis and gain a deeper understanding of workforce instability in this context.

The combination of cross-sectional and longitudinal approaches represents a clear strength of this work. Given the number of models and outcomes examined, several analyses, notably the longitudinal findings, should be considered exploratory, and hypothesis-generating rather than conclusive. In addition, the large number of univariate analyses increases the risk of Type I error, and these results should therefore be interpreted with caution. Accordingly, while the longitudinal component is informative, its results should be interpreted cautiously in light of the small sample size and the possibility of healthy worker bias. The longitudinal subsample was not formally powered for multivariate analyses and should therefore be considered exploratory and hypothesis-generating rather than confirmatory. Larger-scale studies are needed to verify the observed associations. In addition, a potential healthy worker bias cannot be excluded, as participants retained in the longitudinal analyses may represent a healthier and more stable subgroup than those who did not participate at follow-up (left employment, changed profession, retired, or been absent through long-term sickness), which could have led to an underestimation of burnout levels and their evolution over time. In this context, missing data may not have been completely at random, as non-participation could itself be related to burnout severity or work disruption.

However, these results, based on French data, cannot be directly extrapolated to other contexts, given the differences in care and support provided to to individuals with PIMD/polyhandicap across Europe and worldwide (52). A larger-scale multicenter longitudinal study would make it possible to explore this topic in greater depth and thereby strengthen the validity of our findings.

This finding underscores the importance of considering interventions that promote mental well-being and of strengthening strategies aimed at preventing burnout, rather than responding only after it has developed. In our sample, a substantial proportion of participants had fewer than 12 years of education, likely corresponding more frequently to frontline healthcare workers who may have been exposed to distinct stressors and higher levels of stress and burnout during the pandemic (53). Future research should examine whether different professional categories were differentially affected.

Burnout was primarily assessed through the three MBI dimensions—emotional exhaustion, depersonalization, and personal accomplishment—consistent with its multidimensional conceptualization (54). This approach also aligns with the recent systematic review by Baker et al. (9) of burnout among staff working in services for adults with intellectual and developmental disabilities, which reported findings separately for the three MBI dimensions. Dimension-specific analyses therefore allowed potential heterogeneity in the magnitude and direction of associations to be examined across distinct components of burnout. Complementary analyses used a global MBI score to summarize overall burnout severity. Although this approach has been used in some French clinical studies, its use remains debated internationally because combining the three dimensions may obscure their distinct—and potentially divergent—associations. In particular, the interpretation of personal accomplishment differs from that of emotional exhaustion and depersonalization. Results based on the global score should therefore be interpreted cautiously and considered complementary to the dimension-specific findings. Internal consistency was acceptable for emotional exhaustion and personal accomplishment but low for depersonalization (Cronbach’s α = 0.50 at T1 and 0.38 at T2), suggesting limited reliability of this dimension in our sample. This may have reduced the precision of depersonalization-specific associations and calls for caution in their interpretation. Similar low internal consistency for depersonalization (α = 0.46) has been reported in the French validation of the MBI (27). Accordingly, associations observed specifically for depersonalization should be interpreted cautiously.

Mood symptoms were assessed using an *ad hoc* visual analog scale rather than a validated instrument for depression or anxiety (18), which limits the precision of this measure and may have led to some overlap with burnout-related emotional distress. Because burnout and (self-reported) mood disorders share common affective components, the distinction between them is not entirely clear-cut at the measurement level. Our findings should therefore be interpreted with caution, as part of the association between burnout and mood scores may reflect shared variance in emotional distress. The observed association between hospitalization and severe burnout should be interpreted with caution, as hospitalization may represent a cause, a consequence, or simply a marker of burnout severity.

Because individual-level sociodemographic information on non-participants could not be linked to the coordinating center for confidentiality reasons, we were unable to compare respondents and non-respondents on minimal characteristics such as sex, age, and profession. In addition, the reasons for non-participation were not collected, in order to avoid placing pressure on the healthcare workers and to preserve the voluntary nature of participation. This limits the assessment of potential selection bias, although the participation rate remained relatively low and the analytical sample was still sizeable.

In addition, structural and organizational characteristics of the workplace were not taken into account, such as staffing levels, workload, staff-to-patient ratios, shift work, management support, and organizational climate. These unmeasured factors may have influenced burnout and may have contributed to residual confounding. Their role should be investigated in future studies.

## Conclusion

This first study of burnout among healthcare workers caring for patients with PIMD/polyhandicap highlights a high prevalence of severe burnout in this group. Burnout among healthcare professionals is a major public health concern, with important consequences for their well-being and for the quality of care delivered to highly vulnerable patients.

## Data Availability

Authors may not be able to share the data set publicly available for legal or ethical reasons. The data include potentially identifying and sensitive person information. The data are only available upon request from the sponsor (Assistance Publique des Hôpitaux de Marseille, Direction de la Recherche en Santé, 80 rue Brochier, Marseille, France, aap.drs@ap-hm.fr) or the study coordinator Karine Baumstarck, karine.baumstarck@univ-amu.fr).

## Group members of the French Polyhandicap group

Thierry Billette de Villemeur (thierry.billette@aphp.fr), Hôpital Trousseau, Service de Neuropédiatrie - Pathologie du développement, Paris, APHP, France), Marie-Christine Rousseau (co-leader, Hôpital San Salvadour, Hyères, APHP, France), Sherezad Khaldi-Cherif (Union Générale Caisse Assurance Maladie (UGECAM), Ile de France, France), Anne-Emmanuelle Piquet-Massin (Comité d’Études, d’Éducation et de Soins Auprès des Personnes Polyhandicapées, Paris, France), Agnès Felce (Hôpital d’Hendaye, Hendaye, APHP, France), Karine Baumstarck (co-leader, EA 3279, Self-perceived Health Assessment Research Unit, School of Medicine, Aix Marseille Université, France), Pascal Auquier (EA 3279, Self-perceived Health Assessment Research Unit, School of Medicine, Aix Marseille Université, France), Lionel Dany (LPS Laboratory of social Psychology, Aix Marseille Université, France), Any Beltran (EA 3279, Self-perceived Health Assessment Research Unit, School of Medicine, Aix Marseille Université, France), Ilyes Hamouda (EA 3279, Self-perceived Health Assessment Research Unit, School of Medicine, Aix Marseille Université, France), Houria El Ouazzani (EA 3279, Self-perceived Health Assessment Research Unit, School of Medicine, Aix Marseille Université, France), Sibylle Del Duca (EA 3279, Self-perceived Health Assessment Research Unit, School of Medicine, Aix Marseille Université, France), Marie-Anastasie Aim (LPS Laboratory of social Psychology, Aix Marseille Université, France), Narjess Boutalbi (CESAP, France), Isabelle Kemlin (CESAP, France), Julie Roger (CESAP, France), Patrick Julien (CESAP, France), Ponha Heng (Hôpital de La Roche Guyon, Daniel Willocq (Hôpital San Salvadour, Hyères, APHP, France), Maria Valkov (Hôpital San Salvadour, Hyères, APHP, France), Stéphane Pietra (Hôpital San Salvadour, Hyères, APHP, France), Stéphane Lenormand (Hôpital d’Hendaye, Hendaye, APHP, France), Katia Lind (Union Générale Caisse Assurance Maladie (UGECAM), Ile de France.
