# Supplementary material for: Burnout among French healthcare workers caring for individuals with profound intellectual and multiple disabilities/polyhandicap: a complementary cross-sectional and longitudinal study
Source: Front Public Health. 2026 Sep 28;14:1906622. doi: 10.3389/fpubh.2026.1906622 (PMC13656036; doi:10.3389/fpubh.2026.1906622)
Supplement: Supplementary file 1 [file Table_1.docx]

**Supplemental table 1:** Determinants of burnout total score (sample 1)

|  |  | **Burnout total score (MBI total)** | | | | |
| --- | --- | --- | --- | --- | --- | --- |
|  |  | M±SD/R | | p^+^ | β | p^++^ |
| **1-Sociodemographics** | | | | | | |
| **Age** |  | -0.033 | | 0.634 |  |  |
| **Gender** | Male (Ref) | -11.5±15.4 | | 0.818 |  |  |
|  | Female | -10.9±14.1 | |  |  |  |
| **Marital status** | Single (Ref) | -11.6±13.4 | | 0.791 |  |  |
|  | No single | -11.0±14.5 | |  |  |  |
| **Having children** | No (Ref) | -8.6±14.0 | | 0.141 |  |  |
|  | Yes | -11.9±14.4 | |  |  |  |
| **Educational level** | <12 years (Ref) | -10.0±14.5 | | 0.518 |  |  |
|  | ≥12 years | -11.4±14.3 | |  |  |  |
| **Person with handicap** | No (Ref) | -11.3±14.4 | | 0.309 |  |  |
| **at home** | Yes | -7.2±13.6 | |  |  |  |
| **Self-perceived** | Comfortable (Ref) | -12.8±13.9 | | **0.011** | 0.05 | 0.390 |
| **financial status** | Difficult | -7.5±14.7 | |  |  |  |
| **2-Personal health** | | | | | | |
| **Chronic diseases** | No (Ref) | -12.9±13.7 | | **0.012** | 0.08 | 0.220 |
|  | Yes | -7.8±14.8 | |  |  |  |
| **Hospitalization** | No (Ref) | -11.3±14.2 | | 0.266 |  |  |
| **episode** | Yes | -7.7±15.6 | |  |  |  |
| **Mood disorders** | No (Ref) | -13.3±13.2 | | **<0.001** | 0.11 | 0.118 |
| **medications** | Yes | -0.6±14.9 | |  |  |  |
| **3-Professional characteristics** | | | | | | |
| **Care structure** | Specialized rehabilitation center (Ref) | | -11.7±14.3 | 0.313 |  |  |
|  | Residential facility | | -9.6±14.4 |  |  |  |
| **Professional category*** | Technical care (Ref) | -12.1±15.2 | | 0.620 |  |  |
|  | Non-technical care | -10.8±14.3 | |  |  |  |
| **Work schedule** | Full-time (Ref) | -11.2±14.3 | | 0.753 |  |  |
|  | Part-time | -10.3±14.8 | |  |  |  |
| **Seniority in polyhandicap care** (years) | | -0.025 | | 0.724 |  |  |
| **Seniority in current center** (years) | | -0.015 | | 0.834 |  |  |
| **Mood disorders** (score)****** | | 0.511 | | **<0.001** | 0.4 | **<0.001** |
| **Coping strategies***** | Social support | 0.003 | | 0.968 |  |  |
|  | Problem solving | -0.135 | | 0.056 |  |  |
|  | Avoidance | 0.303 | | **<0.001** | 0.20 | **0.002** |
|  | Positive thinking | -0.250 | | **<0.001** | -0.15 | **0.016** |

M±SD: mean ± standard deviation; R: correlation coefficient

Bold values: p-values< 0.05

^+^ p-value univariate analysis; ^++^ p-value multivariate analysis

Linear regression: β standardized beta

* Professional category: Technical Care (nurses, physiatrists, psychometricians, or occupational therapists), Non-technical Care (nurse aids or educators)

** Mood disorders: Scores ranging 0 to 100, the higher the score the higher coping strategy is used

*** Coping strategies (Brief COPE): Scores ranging 0 to 100, the higher the score the higher coping strategy is used

**Supplemental table 2:** Characteristics of the subsample of 58 healthcare professionals included in the longitudinal study (sample 2)

|  |  | **N (%) ; Mean ± SD** |
| --- | --- | --- |
| **1-Sociodemographics** | | |
| **Age** (years) (N=58) |  | 46.7±8.5 |
| **Gender** | Male | 13 (22.4) |
|  | Female | 45 (77.6) |
| **Marital status** (N=57) | Single | 16 (28.1) |
|  | No single | 41 (71.9) |
| **Having children** (N=58) | No | 9 (15.5) |
|  | Yes | 49 (84.5) |
| **Educational level** (N=58) | < 12 years | 25 (43.1) |
|  | ≥12 years | 33 (56.9) |
| **Person with handicap at home** (N=57) | No | 52 (91.2) |
|  | Yes | 5 (8.8) |
| **Self-perceived financial situation** | Comfortable | 35 (61.4) |
| (N=57) | Difficult | 22 (38.6) |
| **2-Personal health** | | |
| **Chronic diseases** (N=58) | No | 32 (55.2) |
|  | Yes | 26 (44.8) |
| **Hospitalization episode** (N=58) | No | 50 (86.2) |
|  | Yes | 8 (13.8) |
| **Mood disorders medications** (N=58) | No | 49 (84.5) |
|  | Yes | 9 (15.5) |
| **3-Professional characteristics** | | |
| **Care structure** (N=58) | Specialized rehabilitation center | 48 (82.8) |
|  | Residential facility | 10 (17.2) |
| **Professional category*** (N=57) | Technical Care | 8 (14.0) |
|  | Non-technical Care | 49 (86.0) |
| **Work schedule** (N=58) | Full-time | 44 (75.9) |
|  | Part-time | 14 (24.1) |
| **Seniority in polyhandicap care** (years) (N=57) | | 16.8±8.5 |
| **Seniority in current center** (years) (N=55) | | 15.9±8.4 |
| **4-Psychobehavorial data** | | |
| **Mood disorders** (score)****** (N=58) |  | 4.8±2.8 |
| **Coping strategies***** | Social support (N=56) | 41.3±21.3 |
|  | Problem solving (N=56) | 56.3±22.6 |
|  | Avoidance (N=55) | 29.0±12.0 |
|  | Positive thinking (N=56) | 55.4±16.2 |
| **Burnout (Maslach Burnout Inventory)** | Total score+ | -10.2±15.9 |
| (N=58) | Emotional exhaustion+ | 24.9±10.7 |
|  | Depersonalization+ | 9.4±4.5 |
|  | Personal accomplishment++ | 44.5±6.7 |

N (%) number (percent)

M ± SD: mean ± standard deviation

^+^ the higher the score the higher burnout level, ^++^ the lower the score the higher burnout level

* Professional category: Technical Care (nurses, physiatrists, psychometricians, or occupational therapists), Non-technical Care (nurse aids or educators)

** Mood disorders: Scores ranging 0 to 100, the higher the score the higher coping strategy is used

*** Coping strategies (Brief COPE): Scores ranging 0 to 100, the higher the score the higher coping strategy is used

**Supplemental Table 3. Factors associated with severe burnout among 58 healthcare workers assessed at 2015 and 2020**

|  |  | **Severe Burnout** | **No severe Burnout** | **p** |
| --- | --- | --- | --- | --- |
|  |  | N (%); M±SD  (N=26) | N (%); M±SD  (N=32) |  |
| **1-Sociodemographics** | | | | |
| **Age** (years) (N=58) |  | 41.0±8.7 | 42.0±8.7 | 0.900 |
| **Gender** (N=58) | Male (Ref) | 9 (34.6) | 4 (12.5) | **0.045** |
|  | Female | 17 (65) | 28 (87.5) |  |
| **Marital status** (N=57) | Single (Ref) | 12 (46.2) | 9 (29.0) | 0.182 |
|  | No single | 14 (53.8) | 22 (71.0) |  |
| **Having children** (N=57) | No (Ref) | 9 (34.6) | 8 (25.8) | 0.469 |
|  | Yes | 17 (65.4) | 23 (74.2) |  |
| **Educational level** (N=56) | <12 years (Ref) | 11 (44.0) | 16 (51.6) | 0.571 |
|  | ≥12 years | 14 (56.0) | 15 (48.4) |  |
| **Self-perceived financial** | Comfortable (Ref) | 1 (3.8) | 1 (3.2) | 0.899 |
| **situation** (N=57) | Difficult | 25 (96.2) | 30 (96.8) |  |
| **2-Personal health** | | | | |
| **Chronic diseases** (N=58) | No (Ref) | 21 (80.8) | 26 (81.2) | 0.963 |
|  | Yes | 5 (19.2) | 6 (18.8) |  |
| **Hospitalization episode** | No (Ref) | 23 (88.5) | 26 (81.2) | 0.451 |
| (N=58) | Yes | 3 (11.5) | 6 (18.8) |  |
| **Antidepressants drugs (last** | No (Ref) | 25 (96.2) | 29 (90.6) | 0.409 |
| **3 months)** (N=58) | Yes | 1 (3.8) | 3 (9.4) |  |
| **3-Professional characteristics** | | | | |
| **Care structure*** (N=58) | SRC (Ref) | 23 (88.5) | 25 (78.1) | 0.300 |
|  | RF | 3 (11.5) | 7 (21.9) |  |
| **Professional category** | Nurse (Ref) | 5 (19.2) | 2 (6.5) | 0.450 |
|  | Nursing assistant | 16 (61.5) | 20 (64.5) |  |
|  | Childcare assistant | 1 (3.8) | 1 (3.2) |  |
|  | Other | 4 (15.4) | 8 (25.8) |  |
| **Work schedule** (N=57) | Full-time (Ref) | 22 (84.6) | 23 (74.2) | 0.336 |
|  | Part-time | 4 (15.4) | 8 (25.8) |  |
| **Seniority in polyhandicap care** (years) (N=57) | | 9.1±6.2 | 13.0±9.8 | 0.173 |
| **Seniority in current center** (years) (N=57) | | 9.7±6.6 | 12.3±9.8 | 0.451 |
| **Mood disorders** (score) (N=56) ****** | | 5.3±2.1 | 4.4±2.4 | 0.101 |
| **Coping strategies** (N=57) ******* | Social support | 46.6±17.1 | 38.3±16.2 | 0.063 |
|  | Problem solving | 52.6±20.0 | 68.5±18.2 | **0.005** |
|  | Avoidance | 30.0±8.6 | 26.6±8.6 | 0.170 |
|  | Positive thinking | 52.1±19.6 | 62.5±17.4 | **0.046** |

N (%) number (percent)

M ± SD: mean ± standard deviation

* Care structure: SRC: Specialized rehabilitation center; RF: Residential facility

** Mood disorders: Scores ranging 0 to 100, the higher the score the higher coping strategy is used

*** Coping strategies (Brief COPE): Scores ranging 0 to 100, the higher the score the higher coping strategy is us
